# The Influence of External Environmental Conditions on Properties of Ceramic Building Materials with Waste Material Additives

**DOI:** 10.3390/ma14112982

**Published:** 2021-05-31

**Authors:** Maria Wesołowska, Anna Kaczmarek, Jerzy Hoła

**Affiliations:** 1 Faculty of Civil and Environmental Engineering and Architecture, University of Science and Technology, 85-796 Bydgoszcz, Poland; anna.kaczmarek@utp.edu.pl; 2 Faculty of Civil Engineering, Wroclaw University of Science and Technology, 50-377 Wroclaw, Poland; jerzy.hola@pwr.edu.pl

**Keywords:** facing walls, ceramic building materials, waste material additives, moisture properties, structure, porosity, mercury intrusion porosimetry

## Abstract

In this paper, we analyze the state of conservation of ceramic building materials (clay masonry units) containing a large share of waste materials in the form of ash and slag from coal combustion and sawdust from wood processing, operated for several decades in facing walls of religious buildings, in external environment conditions. For the purpose of this analysis, comparative tests were carried out on the samples of ceramic materials cut out from facing walls and samples extracted from the same ceramic materials; they were stored in laboratory conditions for the entire time. The following were investigated: initial water absorption, capillary rise, and porosity structure determined with mercury intrusion porosimetry (MIP). The research has shown, among other things, that the ceramic materials exploited in the external environment are characterized by an almost twofold increase in the initial rate of water absorption and by a different size of dominant pores and a pore size distribution in comparison with ceramic materials stored in laboratory conditions. The results, obtained for ceramic building materials (clay masonry units) containing the above-mentioned waste materials in their composition, constitute a novelty. They fill a gap in the literature by establishing how decades-long operation in natural conditions affected the capillary properties and the porosity structure of the ceramics under investigation. Based on the obtained research results, conclusions of cognitive and practical significance have been formulated that relate to the possibility of the exploitation of facing walls made of investigated ceramic materials.

## 1. Introduction

Many buildings, including historic structures, have external facing walls made of traditional ceramic building materials that feature high durability. Facing walls in a significant number of buildings, including religious structures, erected in the second half of the previous century, especially in central Poland, were made of clay masonry units containing a large share of waste materials. At that time, it was one of the solutions for proenvironmental actions in order to reduce the amount of materials on heaps and treated as waste products. These materials include ash and slag from coal combustion and wood processing sawdust.

**Figure 1:**
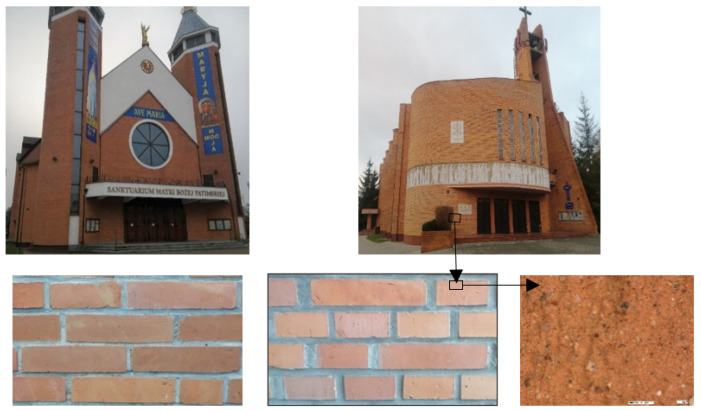
Religious buildings from the second half of the XX century with facing walls made of ceramic building materials with waste material additives from which the test samples were taken.

The ceramic materials featured high surface smoothness (Figure 1), small dimensional deviations, and good quality parameters including relatively high strength.

Therefore, at that time, ceramic building materials were also used for building facing walls, despite the lack of tests and manufacturer’s declarations related to their durability in external environment conditions. It refers to the lack of tests for water absorption and freeze/thaw resistance. Facing walls made of such ceramic building materials are still functioning up to this day, without any clear visual signs of destruction, as documented in Figure 1.

It is important that these walls are protected against capillary rising water from the ground and do not show moisture damage typically caused by water in combination with soluble salts [1,2,3,4,5,6,7].

It should be agreed that a visual assessment of the walls performed after several decades does not provide sufficient information to predict their further durability.

Appropriate research is needed to quantify whether these ceramic building materials have been negatively affected by years of adverse climatic influence and to what extent that situation takes place. However, neither the ways that ceramic building materials currently react to contact with water nor their initial water absorption rates are known. It is also important to know the influence of water on structures, especially on the porosity structure and freeze–thaw cycling of water absorbed during intensive rainfall, its flow on the wall surface, and over several decades of operation in general.

It is worth noting here that there are no limit values of water absorption for ceramic building materials in the literature [8]. Some guidelines in this respect are contained in the study [9], where the maximum amount of water absorption of ceramic masonry units depends on environmental conditions to which the wall made of those units is exposed. Environmental conditions have been defined in [10] by establishing five micro-conditions of exposure classes.

Facing walls made of ceramic building materials in question are exposed to moderate conditions. Therefore, according to [10], the maximum absorption value should not be greater than 18% by weight.

In case of initial rate of water absorption, the limit value is proposed in [9] where it is set at maximum 1.0 kg/(m^2^·min) for clinker masonry units and at 1.8 kg/(m^2^·min) for exterior wall clay masonry units.

When it comes to freeze–thaw cycling of water absorbed by the ceramic building materials, it may cause unfavorable changes in their structure, resulting in shortening the period of their nominal durability. This is mainly a change in pore size distribution, as pointed out by the authors of the studies [11,12,13,14,15]. The references show three basic pore sizes that can be distinguished in ceramic building materials: large, medium, and small. Their initial distribution in the structure of ceramic building materials depends, among other things, on the composition of the molding sand, molding process, and sintering burning temperature [16,17,18]. According to [11,19,20,21,22], large pores of diameters greater than 3.0 µm are beneficial for the freeze–thaw durability resistance of ceramic building materials. In comparison, medium pores of diameters between 3.0 and 0.1 µm are considered to be critical to determine and decide the freeze–thaw cycling resistance [11,23,24,25,26]. Small pores of diameters below 0.1µm where water is freezing at temperatures well below 0 °C [27] are considered to be less important. In addition, as highlighted in studies [19,20,28], the effect of freeze–thaw cycling in ceramic building materials can result in changes of water contact behavior.

Taking the above into account, the aim of this study is to fill the gap in the literature as well as to determine during conducted research how capillary properties and the porosity structure of ceramic building materials containing waste material additives in the form of ash, slag, and sawdust were affected by several decades of operation in natural conditions. The results of the study, which are new and compared with the results obtained for the reference ceramic building materials stored for the same period of time in laboratory conditions, will be of great practical importance. They will provide an answer to the question of whether facing walls made of this type of ceramic materials in many buildings in the second half of the previous century can still be used without any changes or whether they should be treated with surface protection, which, according to [10] is, e.g., plaster coating.

## 2. Characteristics of Materials Used for Production of Tested Ceramic Building Materials (Clay Masonry Units)

For the production of the ceramic building materials that are the subject of this study, we used a molding sand consisting of Pliocene clays with a percentage fraction content as in Table 1 and chemical analysis as in Table 2, ash–slag mixture where particle size analysis is given in Table 3, and wood sawdust.

The composition of the molding sand is depicted in Table 4. The results of chemical analysis of the clays from the period of production of ceramic building materials, presented in Table 2, indicate that they are definitely ferruginous in character and have little marl contamination.

Moreover, the production technology of the ceramic building materials in question consisted of mechanical forming of masonry units using the plastic method and their natural drying, followed by burning in a coal-dust-fired Hoffmann ring furnace at the temperature of 980 °C for 216 h.

Selected quality parameters of tested ceramic building materials are shown in Table 5.

## 3. Description of Testes Samples

**Figure 2:**
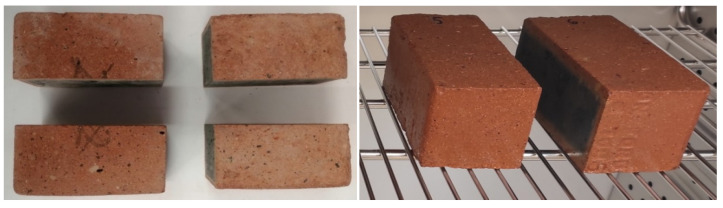
Examples of Group A and B samples prepared for testing.

Samples were obtained and tested from two groups of clay masonry units, designated as A and B, shown as examples in Figure 2.

- Group A consisted of brick samples extracted for testing purposes from facing walls of religious buildings shown in Figure 1, operated in natural outdoor environmental conditions for approximately 35 years. The number of tested samples was 18 and they were chosen randomly. The samples were cut from the wall using an angle grinder, then cleaned in laboratory conditions from any mortar residues and cut to the dimensions of 60 × 120 × 65 mm.
- Group B consisted of brick samples extracted from the same batch of masonry units as group A and stored in laboratory conditions for 35 years at 20 °C (±2 °C) and relative air humidity of 50% (±5%). These masonry units remained in the laboratory as extra units that were obtained during the construction of these buildings in order to perform strength tests. In total, 18 tested samples, also dimensioned as 60 × 120 × 65 mm, were cut from these units.

## 4. Description of the Test

Samples from both groups A and B were tested in the laboratory. First, water absorption by capillary action was assessed, and then water absorption tests were performed. After completion of these tests, porosity tests were performed using mercury intrusion porosimetry (MIP).

### 4.1. Water Absorption by Capillary Action

**Figure 3:**
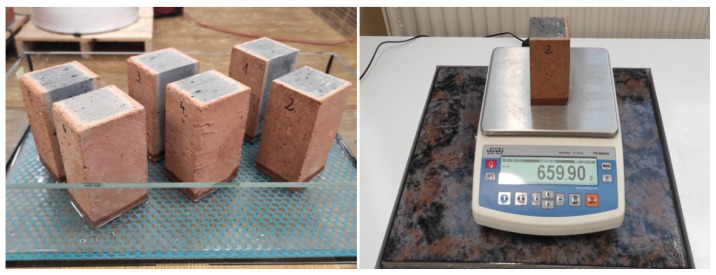
Capillary water rise test stand for samples.

Water absorption by capillary action tests with the samples were carried out according to [29]. The number of tested samples was 18 for each group. The samples were dried to constant weight at +40 °C ± 1 °C and then weighed to the nearest 0.1 g. Drying temperature of 40 °C was used to prevent possible microcracks inside the samples. The samples were placed in a tray on a frame. For testing purposes, sample faces were immersed in water at a constant depth of 5 mm (±1 mm) during testing, as shown in Figure 3. After soaking times of 1, 4, 9, 16, 25, 36, 49, and 64 min, the samples were removed, the surface that was immersed in water was wiped, and the samples were weighed (Figure 3).

Based on the specific mass of water absorbed by the samples, the following was determined:

- initial rate of water absorption, in kg/(m^2^·min)

(1) $$c_{wis}=\frac{m_{so,s}-m_{dry,s}}{A_{s}\cdot t_{so}}\cdot 10^{3},$$

- water absorption due to capillary rise by capillary action, in kg/(m^2^·$\sqrt{min}$)

(2) $$c_{ws}=\frac{m_{so,s}-m_{dry,s}}{A_{s}\cdot \sqrt{t_{s}}}\cdot 10^{3},$$

where:

*m_so,s_*—sample weight after water saturation soaking at time *t*, (g),
*m_dry,s_*—sample weight after drying, (g),
*t_so_*—saturation soakingtime of initial absorption = 1, (min),
*t_s_*—saturation soakingtime, (min),
*A_s_*—sample area of water rise, (m^2^).

### 4.2. Water Absorption Test

**Figure 4:**
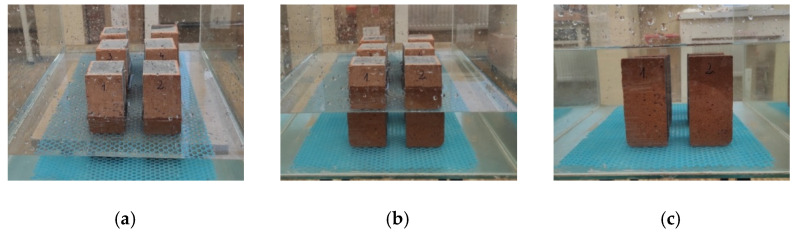
Water soaking stages: (**a**) stage I, (**b**) stage II, (**c**) stage III.

The water absorption test was performed according to [30] by immersion of 18 samples from group A and 18samples from group B. The samples were dried to constant weight at +40 °C ± 1 °C and then weighed to the nearest 0.1 g. A drying temperature of 40 °C was used to prevent possible microcracks inside the samples. The samples were placed in a tray with a frame and immersed in water at the temperature of 20 °C (±2 °C) to about 1/3 of their height. After 3 h, the water level was raised to about 2/3 of the sample height, and after another 3 h, the water was replenished until the samples were fully submerged, as shown in Figure 4.

The samples were kept in water until their constant weight was established. For weighing, samples were taken out individually and drained from water.

Sample absorption nm was calculated from the formula, in %:

(3) $$n_{m}=\frac{C_{m}-C_{s}}{C_{s}}*100,$$

where:

*C_m_*—weight of a sample saturated soaked with water, g,
*C_s_*—weight of a dry sample, g.

### 4.3. Porosity Test Using Mercury Intrusion Porosimetry

**Figure 5:**
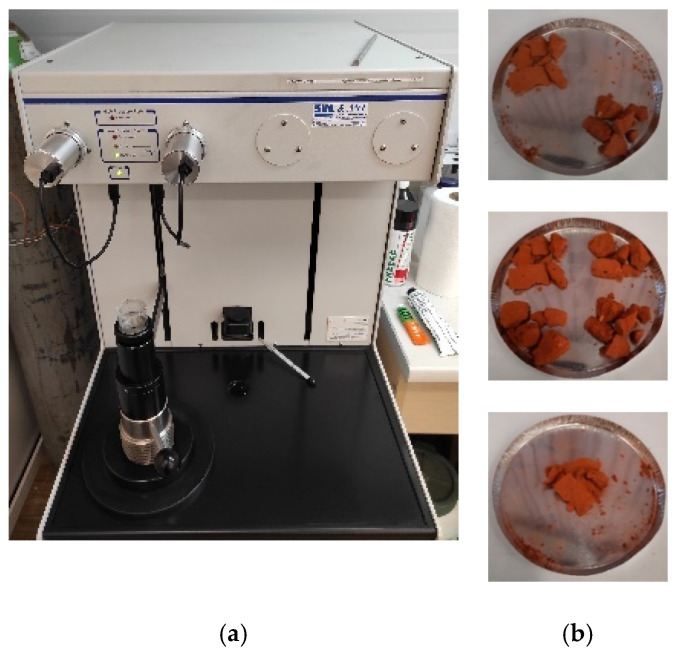
Porosimetry test stand: (**a**) the mercury intrusion porosimeter used in the test, (**b**) samples selected for the test.

For the porosity test, 18 samples from group A and 18 samples from group B were used after the absorption test performed earlier. Fragments of approximately 1 cm^3^ were taken from the facing surface of each sample of both groups. The samples from group A were combined and dried to constant weight at the temperature of 40 °C ± 1 °C. A laboratory sample was selected with the quartering method [31,32] from the obtained material. Quartering consisted of coning the collected and thoroughly mixed material, then flattening and cross-dividing it into four parts. Two diagonal parts were removed, the remaining two parts were remixed, and the selection process was repeated. This procedure was performed three times to obtain laboratory sample volume of about 5cm^3^ that corresponds to the volume of the penetrometer tank shown in Figure 5. The same procedure was used for group B samples.

The test was performed using a 9500 series AutoPore IV mercury intrusion porosimeter equipped with two ports: low and high pressure with a maximum value of 228 MPa, which allows measurements in the range of meso- and macropores from 2 nm to 360 μm (Figure 5a). Before the actual test, the calibration and “blank test” of the penetrometer used in this test were carried out to determine volume, compressibility, and thermal effect. An equilibrium time of 30 s was determined based on control measurements. As a result of the test, the following parameters of the ceramic building material structure in question were determined: total pore volume, sample volume and its skeletal density, the distribution of the pore volume as a function of the pore diameter as integral cumulative, and differential relation.

The share of pore volume (*U*) was calculated based on the relation:

(4) $$U_{nondest}=\frac{\sum_{i>3.0µm}IV_{nondest}}{TIV}\cdot P,$$

(5) $$U_{frost}=\frac{\sum_{i=0.1µm}^{\;\;\;\;\;3.0µm}IV_{frost}}{TIV}\cdot P,$$

(6) $$U_{small}=\frac{\sum_{i<0.1µm}^{}IV_{small}}{TIV}\cdot P,$$

where:

*IV_nondest_*—% share of meso- and macropores larger than 3.0 µm in diameter,
*IV_frost_*—% share of mesopores with diameters in the range of 0.1 to 3.0 µm,
*IV_small_*—% share of nanopores smaller than 0.1 µm in diameter.

## 5. Test Results and Discussion

### 5.1. Water Absorption by Capillary Action

The results of the initial rate of water absorption tests for group A samples extracted from the religious building and group B samples stored in laboratory conditions are given in Table 6. Average values are the average of 18 results.

As seen in Table 6, the average value of initial rate of water absorption for the samples extracted from the building, where they were subject to direct exposure to outdoor climate for about 35 years, is 1.15kg/(m^2^·min). This result is almost double that obtained for samples stored in laboratory conditions. Since the recommended maximum value of initial rate of water absorption for exterior wall masonry units according to [9] is 1.8kg/(m^2^·min), the samples can be considered to meet this requirement.

**Figure 6:**
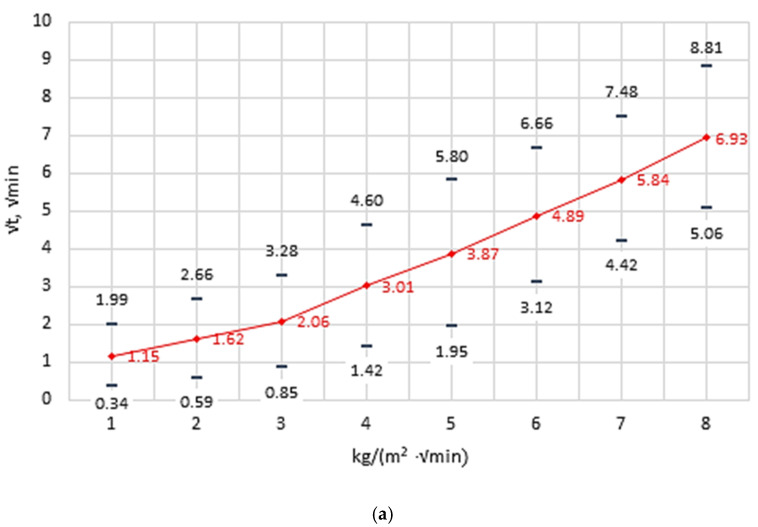

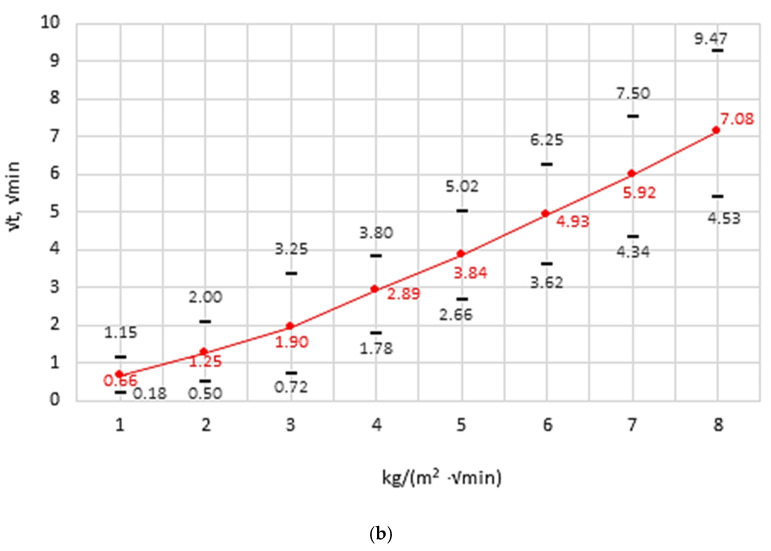
Capillary absorption of tested samples: (**a**) group A samples, (**b**) group B samples.

The results of capillary water absorption tests for group A and B samples are shown in Figure 6. They indicate that the dynamics of capillary water absorption by the samples from both groups is similar.

### 5.2. Water Absorption

The results of absorption tests for group A samples extracted from the religious building and group B samples extracted from the bricks stored in laboratory conditions are given in Table 6. The average values shown are the averages of 18 results.

As seen in Table 6, the average absorption value of the samples extracted from the religious building is 14.55%, which is higher than the value achieved for group B samples by about 1%. Thus, it can be concluded that many years of operation in the natural conditions of the external environment did not significantly affect the increase of water absorption of ceramic building materials in question.

### 5.3. Porosity

**Figure 7:**
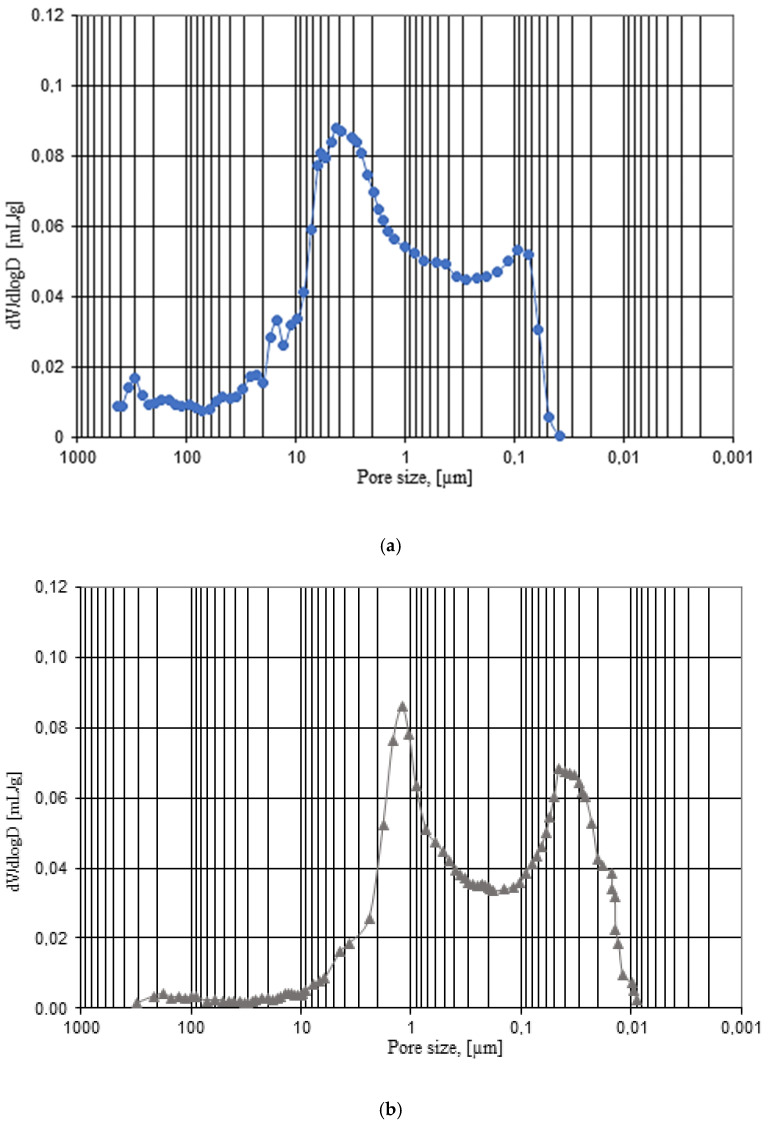
Differential curve of pore size distribution: (**a**) group A samples, (**b**) group B samples.

**Figure 8:**
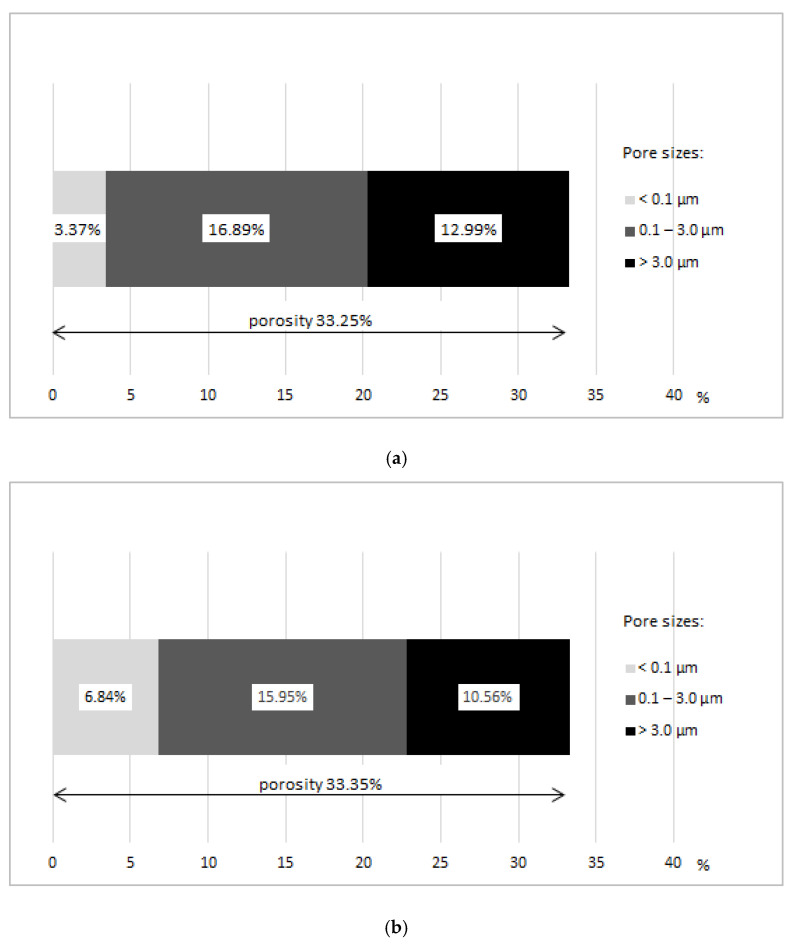
Typical porosity ranges: (**a**) group A samples, (**b**) group B samples.

Test results, obtained through use ofmercury intrusion porosimetry, are given in Table 6 and shown in Figure 7 and Figure 8. The study shows that the samples extracted from masonry units operated for 35 years in outdoor environmental conditions have a total porosity value of 33.35% and a bulk density of 1.668 g/cm^3^. Group B samples stored in laboratory conditions have a similar total porosity value of 33.25% and a bulk density of 1.680 g/cm^3^ to group A samples.

Essential factors that differentiate tested samples from group A and B are dominant pore diameter and porosity structure, the results of which are presented in Figure 7 and Figure 8. In group A samples, after 35 years of exposure to environmental factors, there is a bimodal distribution of porosity with dominant pores of diameters of 3.0 µm and 0.09 µm (Figure 7a). Bimodal porosity distribution is also maintained in group B samples stored in laboratory conditions, with dominant diameters of 1.3 µm and0.045µm (Figure 7b). Obtained dominant diameters above 3µm are, according to [11,23], in the range considered safe from the frost damage point of view.

As seen in Figure 8, samples extracted from the real building (group A) after 35 years of exposure to environmental factors are characterized by total porosity of 33.25%, and the pore proportion in the range of 0.1 to 3.0 µm is 16.89%, above 3.0 µm—12.99%, and below 0.1 µm—up to 3.37%. In case of group B samples stored in laboratory conditions with similar total porosity value of group A samples, the porosity structure is different. Pore share in the range of 0.1 to 3.0 µm is 15.95%, above 3.0µm—10.56%, and below 0.1 µm—6.84%.The obtained results show that as a result of many years of operation, due to freeze–thaw cycling in group A ceramic building materials, the pore content >3 µm was increased, which is, however, beneficial from the point of view of its frost damage resistance. The reduction of pore share content <0.1 µm does not affect frost damage. This pore range is considered safe [11,23]. The pore share in the range of 0.1–3.0 µm considered critical from the point of view of frost damage is similar for both groups, A and B. As a result of environmental exposure over a period of about 35 years, the share of critical pores was increased by less than one percentage point, compared to samples stored in laboratory conditions.

## 6. Summary

In this paper, we present the results of studies on the influence of many years of exposure to external environmental conditions on clay burnt masonry units produced with a large share of waste materials in the form of ash, slag, and sawdust used in the facing walls of religious buildings. These results are a novelty and fill a gap in the literature. Two groups of ceramic samples were tested: bricks that were extracted from a building and bricks that came from the same batch but were stored in laboratory conditions for 35 years. Moisture properties such as initial rate of water absorption, water absorption, and capillary absorption, which are important indicators of brick durability and porosity, were tested using mercury intrusion porosimetry.

It was shown that the average value of initial rate of water absorption for ceramic building materials used for 35 years in natural environment is almost twice as high as for the reference ceramic building materials stored in laboratory conditions. However, it does not exceed the recommended value for ceramic masonry units used in moderate microconditions. It was also shown that the dynamics of water capillary absorption and the average value of absorption for ceramic building material samples extracted from buildings did not change in relation to the samples stored in the laboratory. Based on the study carried out, it may be concluded that long-term exposure to environmental conditions of masonry units with the waste material additives in the form of ash, slag, and sawdust did not cause any significant changes in their moisture properties in relation to reference masonry units. Changes in moisture properties are related to material structure.

The study results of porosity structure obtained using mercury intrusion porosimetry for the group of samples extracted from masonry units embedded in facing walls and operated for 35 years in external environment conditions show that both the value of total porosity and bulk density are similar to the results obtained for the group of samples stored in laboratory conditions. What differentiates the tested ceramic samples of both groups is the diameter of the dominant pores and the porosity structure. Dominant pore diameters are higher for ceramic building materials operated in external environment conditions, for which the pore share of diameters above 3µm was increased. It is true that pores of this size affect higher value of initial water absorption, but they are considered safe from the point of view of frost damage of ceramic building materials, because they are a kind of compensation chamber for stresses related to ice crystallization from frozen water contained in ceramic building materials.

The obtained study results, apart from their cognitive importance and filling the gap in the literature, are of great practical significance. They give the right to claim that facing walls made of these particular ceramic building materials in many religious buildings erected in the second half of the previous century can continue to be used without alteration. Taking the approximate service life of tested structuresinto account, this decision should not be taken unconditionally. Repeating the test after a period of another 15 years has been suggested.

## Figures and Tables

**Table 1:** Aerometric analysis of Pliocene clays used in the production of tested ceramic building materials (clay masonry units).

Fraction Name	Average Content (%)
clay, below 0.002 mm	40.4
silt, from 0.002 to 0.05 mm	36.1
sand, from 0.05 to 2 mm	23.1
gravel, from 2 to 5 mm	0.4

**Table 2:** Chemical analysis of Pliocene clays used in the production of tested ceramic building materials (clay masonry units).

Compound Name	Average Content (%)	Range (%)
SiO_2_	62.60	53.48–70.72
SO_3_	0.31	0.14–0.75
CaO	2.23	0.83–8.02
MgO	1.18	0.60–1.66
Al_2_O_3_	17.06	8.11–22.63
Fe_2_O_3_	5.27	3.44–69.8
Na_2_O	0.64	–
K_2_O	1.97	1.80–2.12
TiO_2_	0.30	–
Loss on ignition	9.40	7.22–14.24

**Table 3:** Particle size analysis of a slag-ash mixture.

Sample No.	Percent Retained on Sieves (%)		
	0.25 mm	0.063 mm	Total
I	6.60	35.50	42.35
II	6.00	34.80	40.90
III	6.45	35.30	41.40
Average value	6.35	35.20	41.55

**Table 4:** The composition of the molding sand used for the production of tested ceramic building materials (clay masonry units).

Raw Materials Name	Molding Sand Share (%)
Pliocene clays	55
Ash–slag mixture	38 (38–40)
Wood sawdust	7 (5–7)

**Table 5:** Selected quality parameters of tested ceramic building materials (clay masonry units).

Quality Parameters	Range	Average Value
compressive strength	16.0–24.0 MPa	19.5 MPa
content of granular marl with more than 0.5 mm fraction	0–0.40%	0.031%
efflorescence of soluble salts	-	none or minimal irremovable bloom

**Table 6:** Summary of test sample results.

Test	Group A	Group B
water absorption initial (kg/(m^2^·min))	0.34–1.99 Average value 1.15	0.18–1.15 Average value 0.66
water absorption (%)	12.44–16.66 Average value 14.55	11.73–15.96 Average value 13.84
general porosity (%)	33.25	33.35
bulk density (g/cm^3^)	1.668	1.680

## Data Availability

Data is contained within the article.
